# Editorial: Heme proteins: key players in the regulation of immune responses

**DOI:** 10.3389/fimmu.2023.1263384

**Published:** 2023-08-09

**Authors:** Claudia Volpi, Benoît J Van den Eynde, Ciriana Orabona

**Affiliations:** ^1^ Department of Medicine and Surgery, University of Perugia, Perugia, Italy; ^2^ Ludwig Institute for Cancer Research, Brussels, Belgium; ^3^ de Duve Institute, Université catholique de Louvain, Brussels, Belgium; ^4^ Walloon Excellence in Life Sciences and Biotechnology, Brussels, Belgium; ^5^ Ludwig Institute for Cancer Research, Nuffield Department of Medicine, University of Oxford, Oxford, United Kingdom

**Keywords:** heme proteins, indoleamine 2,3-dioxygenase, heme oxygenases, immune response, heme

Heme is the iron-containing prosthetic group of heme proteins, with varied functions ranging from gas carriers (i.e., hemoglobin) to metabolic enzymes (i.e., cytochromes, indoleamine dioxygenases). Free heme is scavenged by hemopexin (Hx), and degraded by the activity of the heme oxygenases HO-1 and HO-2. In response to heme-mediated inflammation, cells upregulate the expression of HO-1, an inducible enzyme that degrades heme into biliverdin, carbon monoxide (CO), and iron; biliverdin is subsequently converted to bilirubin. Thus, HO-1 induction functions as a negative feedback mechanism to mitigate the pro-inflammatory effects of heme by reducing its availability (1). Similarly, biliverdin, CO, and bilirubin are also endowed with anti-inflammatory and cytoprotective properties (2). Since free heme is generated by mechanisms of red blood cells damage and tissue injuries, it is considered a danger-associated molecular pattern (DAMP) capable of activating Toll-like receptor 4 (TLR4). Heme-triggered TLR4 can induce the production of pro-inflammatory cytokines, chemokines, and reactive oxygen species (ROS) in various immune cells, such as macrophages, neutrophils, and endothelial cells, promoting the recruitment and activation of further immune cells and causing tissue damage and inflammation (3).

Heme is a well-established pro-inflammatory signaling molecule involved in different pathological settings. Seika et al. elegantly demonstrate that the accumulation of free heme in the colon amplifies the DNA damage, the abnormal proliferation of epithelial cells and can sustain a state of chronic inflammation. This mechanism provides a novel explanation for gastrointestinal syndrome (GIS) and bleeding, two severe gastrointestinal side effects observed during anticancer therapy. Based on this evidence, the Authors propose the depletion of free heme by increasing HO-1 activity and Hx sequestration as a potential therapeutic approach to ameliorate the side effects of common antitumoral therapies (Seika et al.). In this scenario, although the effects on cancer prevention of many antioxidant compounds and food supplements are debated (4, 5), novel therapeutic approaches could be exploited targeting antioxidant heme proteins to ameliorate the side effects of anti-cancer therapies.

Free heme may also exacerbate the inflammatory response during bacterial or viral infections with simultaneous intravascular hemolysis. Thus, the antioxidant effects of bilirubin can be identified as a potential therapeutic tool in a context of high oxidative stress resulting from endotoxemia. Dorresteijn et al. propose that hyperbilirubinemia induced by atazanavir can potentiate the antioxidant capacity and restrain the vascular effects, causing a significant decrease in arterial pressure and preventing vascular hyporeactivity in human systemic inflammation elicited by experimental endotoxemia. These results confirm that a physiologic oxidative state can mitigate a detrimental inflammatory response and this could be beneficial both in cardiovascular diseases, by preventing the progression of vascular dysfunctions, and also in septic patients. As suggested by the Dorresteijn et al., inflammatory and oxidative mechanisms could provide optimal therapeutic targets for sepsis resolution, as the eradication of invading pathogens.

In addition to the proinflammatory effect of free heme, heme-containing enzymes may also play a pivotal role in modulating inflammation and oxidative stress, representing potential therapeutic targets for the treatment of various inflammatory/immune-related diseases. In a murine model of low-dose endotoxemia, Mannarino et al. describe a significant reduction of circulating endothelial progenitor cells (EPCs) caused by the chronic inflammatory condition. Interestingly, the administration of l-kynurenine (l-kyn), the main metabolite of the heme-containing enzyme Indoleamine 2,3-dioxygenase 1 (IDO1), reverted EPC decrease. Accordingly, in patients affected by low-grade inflammation, high level of systemic l-kyn directly correlated to the presence of protective EPCs, suggesting a relationship between the immunomodulatory properties of l-kyn and the number of circulating EPCs. This study also highlights the relevant function of the heme-containing enzyme IDO1 in the control of the pro-inflammatory response in several chronic inflammatory conditions (6–8). Intriguingly, many anti-inflammatory effects exerted by IDO1 in various pathological settings rely on the agonistic activity of its major metabolic product l-kyn on the Aryl Hydrocarbon Receptor (AhR) (9–11). Similarly, both bilirubin and biliverdin can bind the same intracellular receptor and trigger modulatory pathways in different immune cells during innate and adaptive immune responses (12–15). Overall, metabolites generated by different heme-containing enzymes, such as IDO1 or HO-1, could converge into a common mechanism that controls the inflammatory response, thus pinpointing novel potential therapeutic targets for a translational perspective. Furthermore, the heme protein IDO1 represents an appealing target by virtue of its dual nature responsible for its enzymatic and signaling activity. In many tumors, IDO1 represents a key mechanism of acquired immune tolerance in the tumor microenvironment (TME). The IDO1 catalytic activity, by depleting tryptophan and generating immunoregulatory kynurenines, hinders an effective anti-tumor immune response (16–18). Therefore, the inhibition of the catalytic activity of IDO1 represented so far a promising and rational therapeutic strategy for the development of effective antitumoral therapies. Several IDO1 inhibitors reached the clinical trials and epacadostat advanced to the large phase 3 trial ECHO-301/KEYNOTE-252 where its association with pembrolizumab (anti-programmed cell death-1/PD-1 antibody) was evaluated. Unfortunately, while a large piece of pre-clinical evidence supported the use of IDO1 inhibition in the context of immunotherapy in solid tumors (19, 20), the treatment of melanoma patients with the association pembrolizumab/epacadostat failed to improve progression-free survival compared to the monotherapy with pembrolizumab (21). The reasons for the trial failure are various (22) and require a better understanding of IDO1 biology, since IDO1 pathway remains a relevant target in cancer immunotherapy. In the current topic, Panfili et al. describe a further on-target activity of epacadostat demonstrating that, besides the catalytic inhibition of IDO1, it could promote the interaction of IDO1 with molecular partners that mediate the signaling function of IDO1, conferring an immunosuppressive phenotype on plasmacytoid dendritic cells. The role of the signaling activity of IDO1 protein has been recently demonstrated in a murine model of melanoma where it incites the progression of the tumor independently of the catalytic function of IDO1 (23). The dual function of IDO1 relies on different conformations of the protein (i.e., the apo- and holo-IDO1) that are dependent on the intracellular heme availability. Thus, heme could affect the dynamic balance between apo- and holo-IDO1 and thus promote the shift towards the catalytic activity of IDO1.

Overall, the intricate interplay between heme proteins, inflammation, and immune regulation highlights the multifaceted roles of these molecules beyond their traditional functions. This Topic underlines how free heme and heme-containing proteins have emerged as critical modulators of inflammatory responses and immune cell functions. Understanding their mechanisms of action and exploring their therapeutic potential may open up new avenues for developing targeted treatments for inflammatory diseases and immune-related disorders.

## Author contributions

CV: Writing – original draft, Writing – review & editing. BE: Writing – review & editing. CO: Writing – review & editing.
